# Supplementary material for: Characterizing cell-type spatial relationships across length scales in spatially resolved omics data
Source: Nat Commun. 2025 Jan 3;16:350. doi: 10.1038/s41467-024-55700-1 (PMC11699133; doi:10.1038/s41467-024-55700-1)
Supplement: Supplementary file 3 — Supplementary Software 1 [file 41467_2024_55700_MOESM3_ESM.zip › CRAWDAD/plots/supplementary/S7_cluster_corr_heatmap_KSFB_vs_NGPL.pdf]

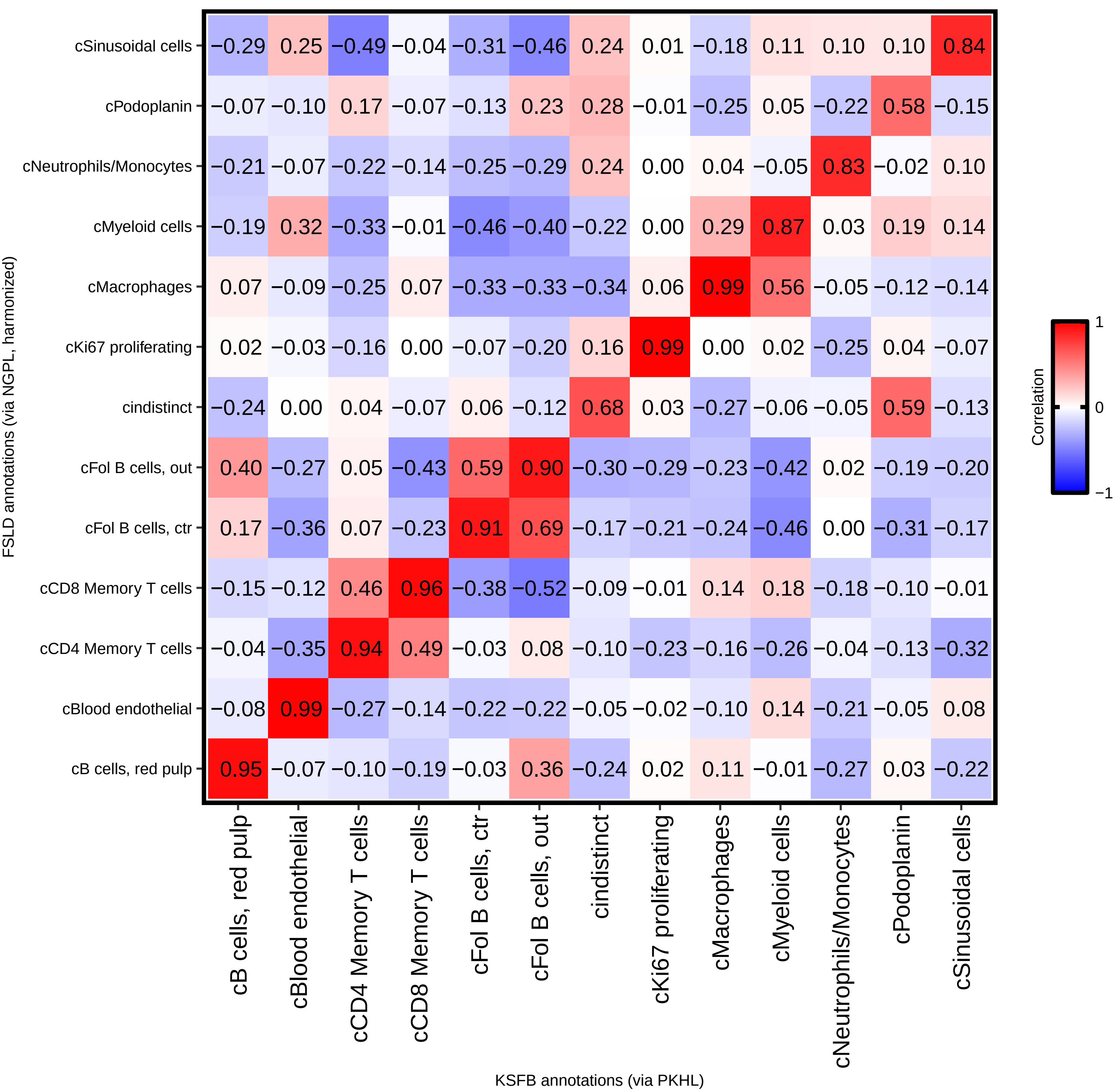

FSLD annotations (via NGPL, harmonized)
cSinusoidal cells −0.29 0.25 −0.49 −0.04 −0.31 −0.46 0.24 0.01 −0.18 0.11 0.10 0.10 0.84
cPodoplanin −0.07 −0.10 0.17 −0.07 −0.13 0.23 0.28 −0.01 −0.25 0.05 −0.22 0.58 −0.15
cNeutrophils/Monocytes −0.21 −0.07 −0.22 −0.14 −0.25 −0.29 0.24 0.00 0.04 −0.05 0.83 −0.02 0.10
cMyeloid cells −0.19 0.32 −0.33 −0.01 −0.46 −0.40 −0.22 0.00 0.29 0.87 0.03 0.19 0.14
cMacrophages 0.07 −0.09 −0.25 0.07 −0.33 −0.33 −0.34 0.06 0.99 0.56 −0.05 −0.12 −0.14
cKi67 proliferating 0.02 −0.03 −0.16 0.00 −0.07 −0.20 0.16 0.99 0.00 0.02 −0.25 0.04 −0.07
cindistinct −0.24 0.00 0.04 −0.07 0.06 −0.12 0.68 0.03 −0.27 −0.06 −0.05 0.59 −0.13
cFol B cells, out 0.40 −0.27 0.05 −0.43 0.59 0.90 −0.30 −0.29 −0.23 −0.42 0.02 −0.19 −0.20
cFol B cells, ctr 0.17 −0.36 0.07 −0.23 0.91 0.69 −0.17 −0.21 −0.24 −0.46 0.00 −0.31 −0.17
cCD8 Memory T cells −0.15 −0.12 0.46 0.96 −0.38 −0.52 −0.09 −0.01 0.14 0.18 −0.18 −0.10 −0.01
cCD4 Memory T cells −0.04 −0.35 0.94 0.49 −0.03 0.08 −0.10 −0.23 −0.16 −0.26 −0.04 −0.13 −0.32
cBlood endothelial −0.08 0.99 −0.27 −0.14 −0.22 −0.22 −0.05 −0.02 −0.10 0.14 −0.21 −0.05 0.08
cB cells, red pulp 0.95 −0.07 −0.10 −0.19 −0.03 0.36 −0.24 0.02 0.11 −0.01 −0.27 0.03 −0.22
cB cells, red pulp
cBlood endothelial
cCD4 Memory T cells
cCD8 Memory T cells
cFol B cells, ctr
cFol B cells, out
cindistinct
cKi67 proliferating
cMacrophages
cMyeloid cells
cNeutrophils/Monocytes
cPodoplanin
cSinusoidal cells
KSFB annotations (via PKHL)
Correlation
1
0
−1
